# The International Medical Annual and Practitioner’s Index

**Published:** 1891-05

**Authors:** 


### BOOK NOTICES.

The International Medical Annual and Practitioners' Index. A Work of Reference for Medical Practitioners. Ninth year, 1891. New York: E. B. Treat, 5 Cooper Union. Price, $2.75.

This excellent work is a record, in a single handy volume, of the progress made in medicine during the year 1890, past.

It is arranged in a series of chapters, and the subdivisions of the chapters are arranged in alphabetical order; the principal word of the subdivision constituting its name being printed in bold type. The index to the whole volume is very thorough, and contains three thousand references to diseases and remedies.

That our readers may form a better idea of the character of the book, we quote the titles of a few of the chapters: "The Hand as a Diagnostic Factor in Diseases of the Nervous System," "The Character of the Sputum as an Aid to Diagnosis," "Dictionary of New Treatment," "Sanitary Science," "Concerning Climatology and Hygiene," "Improvements in Pharmacy." As a point of special interest to homœopathists, we will mention that among the new remedies appears Cactus grandiflorus, and the credit of introducing it to the profession as a remedy for heart troubles is placed where it belongs, with Dr. Rocco Rubini, of Naples. Its great characteristic or keynote—*sensation as if the heart were constricted by an iron hand*—is given in italics.

This is given as a slight illustration of the value of the book.

